# Intradialytic Hypotension and Cardiac Remodeling: A Vicious Cycle

**DOI:** 10.1155/2015/724147

**Published:** 2015-01-14

**Authors:** Chia-Ter Chao, Jenq-Wen Huang, Chung-Jen Yen

**Affiliations:** ^1^Renal Division, Department of Internal Medicine, National Taiwan University Hospital, Jin-Shan Branch, 51 Nan-Shih, Jin-Shan District, New Taipei City 208, Taiwan; ^2^Graduate Institute of Toxicology, National Taiwan University Medical School, Section 1, 1 Jen-Ai Road, Zhong-Zheng District, Taipei 100, Taiwan; ^3^Renal Division, Department of Internal Medicine, National Taiwan University Hospital, 7 Chung-Shan South Road, Zhong-Zheng District, Taipei 100, Taiwan; ^4^Department of Geriatrics and Gerontology, National Taiwan University Hospital, 7 Chung-Shan South Road, Zhong-Zheng District, Taipei 100, Taiwan

## Abstract

Hemodynamic instability during hemodialysis is a common but often underestimated issue in the nephrologist practice. Intradialytic hypotension, namely, a decrease of systolic or mean blood pressure to a certain level, prohibits the safe and smooth achievement of ultrafiltration and solute removal goal in chronic dialysis patients. Studies have elucidated the potential mechanisms involved in the development of Intradialytic hypotension, including excessive ultrafiltration and loss of compensatory mechanisms for blood pressure maintenance. Cardiac remodeling could also be one important piece of the puzzle. In this review, we intend to discuss the role of cardiac remodeling, including left ventricular hypertrophy, in the development of Intradialytic hypotension. In addition, we will also provide evidence that a bidirectional relationship might exist between Intradialytic hypotension and left ventricular hypertrophy in chronic dialysis patients. A more complete understanding of the complex interactions in between could assist the readers in formulating potential solutions for the reduction of both phenomena.

## 1. Introduction

Intradialytic hypotension (IDH) is a common scenario familiar to both patients under chronic hemodialysis and nephrologists. It is defined as a decrease in systolic blood pressure (BP) by more than 20 mmHg or decrease in mean arterial pressure (MAP) by 10 mmHg, accompanied by symptoms including (but not limited to) abdominal discomfort, yawning, sighing, nausea/vomiting, muscle cramping, restlessness, dizziness/fainting, or anxiety [1]. The incidence of IDH during each session of hemodialysis lies in somewhere between 20% and 30% [1–3]. IDH, in the short term, impairs patients' quality of life by causing nuance symptoms and creates barriers to achieving adequate dialysis dose and ultrafiltration, while in the long term it leads to cardiovascular complications (ischemic heart events, arrhythmia), more hospitalization, and higher mortality [4, 5].

## 2. Development of IDH

Risk factors for IDH have not been defined clearly from the literature, but several issues have been held responsible. For example, demographic backgrounds and medical comorbidities play an important role in determining IDH susceptibility. Patients of advanced age (≧65), with diabetic nephropathy, cardiovascular illnesses, or autonomic dysfunction, are at risk for developing IDH [1, 6–8]. Furthermore, clinical features such as low blood pressure levels before hemodialysis session (<100 mmHg), poor nutritional status (hypoalbuminemia), or severe anemia also predispose chronic dialysis patients to IDH occurrence [9]. Among these risk factors, cardiovascular comorbidities, whether present before or after maintenance dialysis initiation, serve as an important step stone for the development or the perpetuation of subsequent IDH.

Most researchers concur that IDH frequently occurs during dialysis, when large volumes of fluid are removed within one session. Rapid ultrafiltration then fails to elicit compensatory cardiovascular (CV) responses, such as vasoconstriction and rising cardiac output, while the combination of inadequate peripheral vascular tone and plasma refilling insufficiency leads to the drop of BP [3]. Clearly, four counteractive components are essential for the body's defense against IDH, that is, cardiac chronotropy and inotropy, plasma refilling, passive venoconstriction, and active arterioconstriction [3, 10]. Effective rise in heart rate in response to IDH could be an important component for countering acute hypotension. Among these four factors, the changes in heart function seem to be the major ones comparing with the others, since the legacy of dysfunctional cardiac machinery could last even after dialysis sessions completion [11], while other factors would not. Consequently, we will discuss in the following the potential role of cardiac remodeling in end-stage renal disease (ESRD) patients and its relevance with IDH. Furthermore, we will also address the issue that IDH might also lead to cardiac changes, contributing to an ultimate downward spiral.

## 3. Cardiac Alterations in ESRD

### 3.1. Left Ventricular Hypertrophy (LVH)

For patients with chronic kidney disease (CKD), the prevalence of LVH increases progressively as renal function deteriorates, accompanied by an elevation of pulse pressure [12]. Nearly three-fourths of chronic dialysis patients have LVH, based on echocardiographic findings [13–15]. These facts suggest that cardiac structural alterations have already taken place during early renal function impairment, and the condition worsens further after dialysis initiation [16]. Advanced age, hypertension, and increased arterial stiffness are significant predictors for LVH in general population and dialysis patients [13, 17]. The pathogenic interplay of LVH of dialysis patients encompasses three factors: high afterload, high preload, and miscellaneous factors, such as renal failure* per se* [16, 18]. The presence of LVH in dialysis patients correlates significantly with subsequent CV events, with a dose-response relationship. Zoccali et al. identified that dialysis patients with LVH had a 2-3-fold higher mortality than those without [19]. Specifically, every 1 g/m^2^/month increase in LV mass index could lead to a 62% increase in the risk of CV events, applicable to both concentric or eccentric types [19, 20]. Similarly, every 1 gram of LV mass reduction could translate into 1% CV risk decrease [21]. This association between LVH and CV risk is potentially mediated by lethal atrial or ventricular, but the exact pathway remains elusive [22–25].

### 3.2. Left Atrial Enlargement

As left atrium (LA) is positioned to sustain pressures from LV diastole, LA sizes/functions are often touted as an indirect indicator of diastolic function [26]. LA volume index (LAVI) is identified as a morphologic surrogate for LV diastolic dysfunction and an indicator for subsequent cardiac remodeling [27, 28]. For dialysis patients, studies on LA functional/structural changes are far fewer [29]. Barberato and colleagues found that 37% of dialysis patients had LAVI higher than 35 mL/m^2^, while others identified that ESRD patients have a progressive increase in LA sizes over time (11% over 1.5 years) [30, 31]. Furthermore, LA volumes changes over time are predictive of incident CV events in dialysis patients, independent of LV mass index [32, 33]. Consequently, the alterations of LA in dialysis patients represent another underestimated issue.

### 3.3. Atrial Fibrillation (Afib)

Cardiac rhythmic abnormalities, especially Afib, occur in 7–27% of all ESRD patients and increase over higher dialysis vintage [34–36]. Thromboembolic events (cerebrovascular accidents) and the preload reduction from Afib might precipitate hemodynamic alterations and myocardial ischemia, leading to adverse prognosis [34]. Development of Afib in dialysis patients is associated with more hospitalization, CV events, and poorer survival [34]. Incident Afib also impairs renal prognosis in CKD patients [37].

## 4. From Cardiac Remodeling to IDH: The Road In Between

Most patients, when they reach CKD stage 5, already carry a moderate to severe burden of CV illnesses, whether from the morbidities that cause their CKD (DM, hypertension) or from the imbalances of divalent ion homeostasis (CKD-MBD, mineral bone disease). The remodeling processes found during initiation of dialysis could simply be the trails left by the past injuries or the compensatory mechanisms. Among these, cardiac remodeling is the predominant manifestation and could contribute mechanistically to hemodynamic instability. Moreover, the sequels of cardiac remodeling might actively contribute to the development of complications during dialysis, most important of all, IDH. It would then be necessary to discuss the role of cardiac remodeling, especially LVH, in the promotion of IDH from two different points of view, that is, the passive one and the active one.

### 4.1. LVH Sets the Background for IDH Development

First of all, LVH plays a permissive role in the development of IDH. ESRD represents a combination of volume overload (fluid retention, the presence of arteriovenous shunt, anemia, etc.) and pressure overload (increasing arterial stiffness, atherosclerosis, etc.). However, the geometry of LV in ESRD mostly presents as concentric hypertrophy owing to pressure overload (from increased systemic resistance), despite the concurrent existence of significant volume component [38, 39]. Those with eccentric hypertrophy (from increased systolic and diastolic wall stress), on the contrary, are accompanied by coronary artery diseases, causing LV dilatation and later systolic dysfunction [40]. These mechanical/morphologic subtypes (concentric versus eccentric) each characterize different response patterns to fluid removal during hemodialysis.

### 4.2. Concentric LVH

Current literature attributes the predisposing effect of concentric LVH on IDH to both hemodynamic factors and structural factors, such as valvular degeneration. First, for patients with concentric LV, they are particularly sensitive to abrupt changes in cardiac loading statuses, predisposing them to prominent BP fluctuation during ultrafiltration [41]. This phenomenon stems from the fact that LV stiffening and reduction in compliance is associated with elevation of end-diastolic pressure. As filling of LV becomes difficult during dialysis (decreasing circulating volume), even low levels of volume reduction could translate into wide variation in cardiac output and then BP. This has been found to be the major etiology of LVH-related IDH, that is, the diastolic dysfunction theory [3]. Also, concentric hypertrophy is often associated with progressive myocardial fibrosis [42], further enhancing LV stiffness and the downstream adverse sequences. Second, the presence of LVH is frequently associated with the occurrence of aortic stenosis, as well as higher myocardial injury, represented by elevated plasma cardiac troponin I [43]. Prolonged aortic stenosis could expectedly lead to hemodynamic instability, precipitating IDH episodes.

### 4.3. Eccentric LVH

Eccentric hypertrophy, on the other hand, is rarely discussed regarding its impact on IDH. Nonetheless, we propose two potential explanations linking eccentric LVH and IDH occurrence. First of all, systolic failure evolving from persistent eccentric LVH could be responsible. Reportedly eccentric LVH demonstrates less influence on IDH comparing with the concentric type, owing to their preservation of LV cavity and the correspondingly higher LV volume in the early phase of cardiac remodeling [40]. This minute advantage vanishes with the development of pump failure later, and the rapid change in loading statuses during ultrafiltration will eventually lead to IDH in these patients. In this instance, systolic heart failure would be the main working hose behind the scene of IDH. Second, flow-mediated obstruction could also assist in IDH precipitation. Indeed, eccentric hypertrophy is often accompanied by asymmetric septal hypertrophy (ASH), caused by sympathetic surge during ultrafiltration processes during dialysis [44]. ASH, with the resultant sympathetic mediated hypercontractility of the hypertrophic myocardium, might cause midsystolic obstruction at the level of LV outflow tract, serving as another pathway to IDH [44]. Furthermore, tachycardia caused by sympathetic output might also contribute to IDH with preload reduction.

### 4.4. LVH Proactively Induces IDH Occurrence

Finally, LVH actively contributes to IDH occurrence, through the induction of myoischemia and arrhythmia. As the hypertrophied myocardium has to work against higher systemic resistance during LVH progression, the oxygen consumption rate increases and sets the stage for ensuing ischemic myocardial damages. This LVH-associated ischemia is often caused by coronary microvascular dysfunction, which is a common scenario in dialysis patients [45]. Furthermore, the reduction in cardiac output from LV filling difficulty will drive the heart rate higher for compensation purpose, while the accompanied tachycardia also elevates myocardial oxygen demand. These ischemic changes could impair myocardial performance and further lead to fibrosis.

Fatal/nonfatal arrhythmia could be another problem in the LVH process. Myocardial ischemia from LVH could enhance the arrhythmogenicity and potentially increase the chance of IDH [46]. LVH* per se*, by means of prolongation of action potential duration and refractory period, along with inherent nonuniform property within the involved myocardium, also potentiates the proarrhythmic phenotype [47]. Both factors could be important mediators for IDH episodes.

## 5. IDH: Also a Precipitant for Cardiac Remodeling?

Few researchers, if present, have addressed the issue of IDH and its contribution to cardiac remodeling, besides its well-known effect on long-term fistula outcomes. However, a diverse spectrum of putative causal relationships could exist between IDH and cardiac remodeling, especially LVH. We would focus more on the occult effect of IDH in the development of LVH in the following section.

### 5.1. Coronary Hypoperfusion and Myocardial Stunning Theory

Intuitively, IDH causes systemic hypoperfusion, including coronary vessels. This ischemic effect, though transient, could potentially cause myocardial fibrosis in the long term and the subsequent LV kinetic dysregulation. A small-scale study using continuous intradialytic hemodynamic monitor did show that IDH or intradialytic muscle cramping correlated significantly with lower cardiac index and higher peripheral resistance [48]. This myocardial injury applies to both ventricle and atrium, resulting in LA dilatation and LVH alike [30]. The combination of coronary hypoperfusion and resultant myocardial dysfunction culminates in an elevated mortality in chronic dialysis patients. In addition, the intermittent reduction in fluid removal due to IDH also predisposes patients to chronic volume overload and eventually myocardial remodeling.

Furthermore, intermittent IDH produces a state called “HD-induced myocardial stunning” [49], which is caused by repeated ischemia and excessive ultrafiltration. Originally described as a delayed recovery of regional myocardial contractile function after reperfusion despite the absence of irreversible damage, the role of myocardial stunning has been subsequently affirmed in chronic hemodialysis patients, as a prelude of heart failure. Myocardial stunning is usually characterized by advanced age, higher ultrafiltration volumes, elevated cardiac troponin I, and, most importantly, presence of IDH [49]. Importantly, impaired calcium regulation, endothelial dysfunction, and the reperfusion period after IDH, which generates free radicals within myocardium, all contribute to this phenomenon [50]. The presence of myocardial stunning is associated with long-term adverse effects on LV function and morphology, contributing to the occurrence of heart failure, while repeated IDH serves as a predictor of poorer long-term outcomes [49].

### 5.2. Neurohumoral Activation Theory

Several studies have found that IDH-prone patients have higher baseline plasma angiotensin II levels than those who are IDH-resistant, and activation of the renin-angiotensin system (RAS) is known to be involved in LVH development, independent of afterload [51–53]. Patients with frequent IDH also demonstrate higher levels of immune activation, manifesting as higher interleukin-6 and C-reactive protein (CRP) comparing with those without [54]. The elaboration of cytokine in this circumstance also carries the potential of promoting LVH [51]. Indeed, cytokine surge and chronic inflammation could cause erythropoietin hyporesponsiveness, with the resultant anemia, inadequate myocardial oxygen supply, and cardiac remodeling. Furthermore, IDH often occurred in the context of dry-weight probing and intense ultrafiltration, which constitutes a strong sympathetic stimulator and RAS activation.

### 5.3. Nutritional Deficiency Theory

IDH could cause profound disturbance in dialytic clearance and reduce the ultrafiltration efficiency in chronic dialysis patients. The poorer urea clearance might lead to azotemic symptoms, and the accompanied malnutrition statuses may have devastating influences. Lack of iron supplement, or even L-carnitine, could predispose patients to anemia, and worsening of anemia would lead to pressure overload and contribute to subsequent LVH progression [55]. Likewise, deficiency of vitamin D in dialysis patients might as well have immunologic consequences and activate intracardiac RAS, which is correlated with LVH development [56, 57].

**Figure 1:**
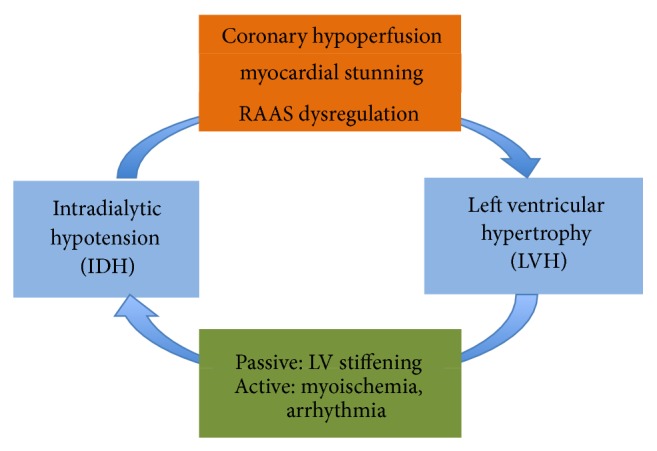
Diagram illustrating the interaction between IDH and LVH. IDH, intradialytic hypotension; LVH, left ventricular hypertrophy; RAAS, renin-angiotensin-aldosterone system.

In summary, from these findings, multiple pathways in dialysis patients might link the occurrence of IDH with the susceptibility to LVH development or perpetuation (Figure 1).

## 6. Manage IDH and Ameliorate LVH Concurrently

Currently, there are several measures commonly adopted to reduce the incidence of IDH, particularly dialytic techniques. A cautionary approach to dietary salt and possibly fluid intake should be recommended for IDH-prone patients, since lower interdialytic weight gain could decrease the likelihood of developing IDH. Fasting during dialysis session is also effective in combating IDH.

Anecdotal reports identified the utility of certain pharmacologic approach for IDH reduction. In a pilot study, researchers compared the efficacy of continuous vasopressin infusion with normal saline infusion on the incidence of fixed-ultrafiltration induced symptomatic IDH [58]. They found that IDH occurred significantly more frequently in the control group. Others administered carnitine in the hope of reducing intradialytic muscle cramps and IDH frequency, but a meta-analysis concluded that carnitine was ineffective [59].

Dialysate adjustment or dialytic regimen titrations are the most common means utilized for reducing IDH. Strategies including ultrafiltration modeling and sodium profiling have been demonstrated to maintain hemodynamic stability better than traditional settings. A study with a modest case number showed that patients receiving a combination of sodium-profiled and ultrafiltration-profiled dialysis presented fewer episodes of IDH than those without such treatment [60]. Both high dialysate bicarbonate and calcium concentrations could improve the hemodynamic patterns for dialysis patients. Compared with conventional bicarbonate-based dialysis, acetate-free biofiltration effectively lowered the frequency of IDH as well.

In addition, newer generation of technology also carries the potential of improving hemodialysis course. Hemodiafiltration has been found to outperform traditional HD regarding its superior middle molecule clearance, and the convective therapy seems to exert beneficial effect on IDH reduction as well [61]. Another treatment, termed as automatic biofeedback-controlled dialysis system, permits the adjustment of dialysate concentration and ultrafiltration settings according to body weight and sodium content. Several randomized trials assessing the biofeedback-based control system disclosed significant reduction in the frequency of IDH and nursing interventions among users [62, 63]. Furthermore, the use of automated biofeedback dialysis significantly reduced reversible LV regional wall motion abnormalities [64]. This undoubtedly solidifies the intertwining relationship between IDH and cardiac modeling.

## 7. Conclusion

IDH and myocardial remodeling, especially LVH, are closely associated with each other. LVH is an important determinant and etiology of IDH, as LVH not only paves the way toward frequent IDH occurrence but also actively facilitates the drop of BP during dialysis, through mechanisms including arrhythmia and myocardial ischemia. Although IDH might not be the predominant cause of LVH in dialysis patients, it represents one potentially remediable factor, in light of the current trend that focuses on treating LVH* per se* for ESRD patients. It is truly difficult to disentangle the relationship between each other, but a clearer understanding of the complex interactions between IDH and LVH might assist in devising useful strategies to avoid the occurrences of both.
